# But I Was So Sure! Metacognitive Judgments Are Less Accurate Given Prospectively than Retrospectively

**DOI:** 10.3389/fpsyg.2016.00218

**Published:** 2016-02-19

**Authors:** Marta Siedlecka, Borysław Paulewicz, Michał Wierzchoń

**Affiliations:** ^1^Consciousness Lab, Institute of Psychology, Jagiellonian UniversityKrakow, Poland; ^2^University of Social Sciences and HumanitiesKatowice, Poland

**Keywords:** metacognition, metacognitive awareness, confidence rating, decision-making

## Abstract

Prospective and retrospective metacognitive judgments have been studied extensively in the field of memory; however, their accuracy has not been systematically compared. Such a comparison is important for studying how metacognitive judgments are formed. Here, we present the results of an experiment aiming to investigate the relation between performance in an anagram task and the accuracy of prospective and retrospective confidence judgments. Participants worked on anagrams and were then asked to respond whether a presented word was the solution. They also rated their confidence, either before or after the response and either before or after seeing the suggested solution. The results showed that although response accuracy always correlated with confidence, this relationship was weaker when metacognitive judgements were given before the response. We discuss the theoretical and methodological implications of this finding for studies on metacognition and consciousness.

## Introduction

When realizing we were wrong we sometimes disappointedly think: “But I was so sure!” We remember being certain that we would give the right answer or make the correct choice. But how well does confidence predict future decision accuracy? Is certainty in a forthcoming response as accurate as certainty in a response that has been already given? In this article we present an experiment aiming to answer these questions by comparing metacognitive accuracy of prospective and retrospective confidence judgments.

The term “metacognition” most generally means “cognition about cognition” and refers to knowledge about ongoing task performance (Fleming and Dolan, 2012), access to one's knowledge (Scott and Dienes, 2008), perceptual awareness (Rounis et al., 2010), or even self-awareness (Metcalfe and Son, 2012). Metacognition has been studied in various research fields such as metamemory (e.g., Busey et al., 2000), decision-making (e.g., Pleskac and Busemeyer, 2010), and metacognitive awareness (e.g., Sandberg et al., 2010). The common issue in those studies is the assessment of metacognitive accuracy (also referred to as metacognitive sensitivity or performance), meaning the extent to which metacognitive judgments predict the accuracy of task performance (also called type 1 task). Across fields, many measures have been introduced (see e.g., Fleming and Dolan, 2012) which assess metacognition at different time points in relation to a type 1 response. However, the potential difference in metacognitive accuracy measured retrospectively and prospectively is often not taken into consideration. For example, metacognitive awareness is measured sometimes before (e.g., Del Cul et al., 2009; Wierzchoń et al., 2012, 2014; Jachs et al., 2015), sometimes after (e.g., Del Cul et al., 2007; Wierzchoń et al., 2012, 2014; Zehetleitner and Rausch, 2013; Jachs et al., 2015) and sometimes simultaneously with a response to a type 1 task (e.g., Rounis et al., 2010).

Retrospective and prospective judgments have been differentiated and studied extensively by metamemory researchers. While prospective judgments are used to predict future performance, retrospective judgments refer to the accuracy of past responses. Prospective metacognition is typically measured either at the stage of acquiring knowledge or at the time of retrieval. For example, participants might be asked to predict the probability of future recall of an item they have just studied, that is, to give judgments about learning (JOL; Dunlosky et al., 2005). Participants might also be presented first with test questions and, when not able to retrieve the answer, asked to judge the probability of recognizing it later, that is, to rate their feeling of knowing (FOK; Metcalfe et al., 1993). Retrospective metacognition has been usually measured as person's confidence in a given response, for example, recognizing a stimulus presented earlier (Busey et al., 2000). It is important to note that when both prospective and retrospective judgments are given in the same study, participants are usually first asked to recall or recognize items presented previously and then to assess confidence in their responses. Then, feeling of knowing is reported only for items that were not recalled correctly (Schnyer et al., 2004). Similarly, judgments of learning are sometimes taken after retrieval attempt and after confidence ratings (Dougherty et al., 2005). Therefore, although FOKs and JOLs are prospective in a way they refer to future performance, they might be, similarly to retrospective judgements, reported after initial, pre-judgment response.

Although both prospective and retrospective judgments correlate with actual performance accuracy (e.g., Chua et al., 2009), to the best of our knowledge, the accuracy of metacognitive judgments immediately preceding and following type 1 responses has not been directly compared in the same study. Such a comparison seems interesting concerning different neural correlates of prospective and retrospective judgments (Schnyer et al., 2004; Pannu et al., 2005; Chua et al., 2009), but also is crucial in the context of the on-going debate about mechanisms of metacognition. Two main theoretical proposals have been offered by memory researchers. The first, called “direct-access” or “trace-strength,” states that metacognitive judgments are based on direct (but sometimes only partial) access to memory content that allows people to know that they know, even though they cannot recall (i.e., articulate) given information at the moment (Hart, 1965; Metcalfe, 2000). The alternative, “cue-utilization” view, states that metacognition is a result of conscious and unconscious inference that might be based on number of cues derived from knowledge and experience (e.g., Koriat and Levy-Sadot, 2000; Serra and Metcalfe, 2009). For example, to judge their future performance in a task, people use general knowledge about their memory functioning and their previous experience with a given type of content (Koriat and Levy-Sadot, 2000). Metacognitive judgments might be additionally informed by cognitive experiences like retrieval fluency or item familiarity (Metcalfe et al., 1993; Koriat and Ma'ayan, 2005).

The quest for a more general explanation of metacognitive judgements has been undertaken in the area of studying the decision-making process and judgment confidence. Here, similarly to the metamemory studies, attempts are made to explain the basis of metacognitive judgments, but on a much shorter time scale. Most contemporary confidence theories are based on the assumption that each decision and judgment is a result of accumulating evidence over time. However, certainty theories differ in terms of whether the evidence available at the time of a type 1 decision (i.e., match between test item and memory trace) is the only information fed to confidence. On one hand, the “direct translation hypothesis” (Higham et al., 2009; Fleming and Dolan, 2012), derived from signal detection theory, states that both confidence judgment about a given choice and the choice itself are based on the same information (i.e., Vickers and Lee, 1998). Therefore, similarly, with regards to the “trace-strength” view on memory, this should imply no differences between the accuracy of retrospective and prospective confidence judgments. The alternative view states that metacognitive accuracy depends on additional, post-decision processing (Petrusic and Baranski, 2003; Pleskac and Busemeyer, 2010). This, similarly to the inference account, allows the possibility that retrospective confidence judgments are based on a different amount of information than prospective ones.

It is important to note that confidence is usually defined as a retrospective judgment. Although many certainty models have been proposed they do not explain prospective confidence as they apply to tasks in which a metacognitive judgment is given either after a type 1 response (i.e., Pleskac and Busemeyer, 2010) or at the same time (i.e., “sure the word was presented previously”; Ratcliff and Starns, 2009). However, the results of neurophysiological and behavioral studies on perception and memory suggest that some results of post-decisional processing could be available to retrospective judgments but not to prospective judgments and type 1 response. For example, it has been shown that in speeded response tasks stimuli-related information is still processed when a type 1 response is being executed, even though the amount of evidence does not change (Burk et al., 2014). Therefore participants can realize they are committing errors and change their responses when allowed (Van Zandt and Maldonado-Molina, 2004; Resulaj et al., 2009; Burk et al., 2014). Making a mistake decreases participants' confidence. The error-related electroencephalography activity, present after an erroneous motor response has been launched, is associated with lower confidence in the preceding perceptual decision (Scheffers and Coles, 2000; Boldt and Yeung, 2015). Moreover, a level of certainty is related to the amount of time it takes to prepare and execute a type 1 response. Shorter reaction time in a perceptual task is associated with increased confidence (i.e., Petrusic and Baranski, 2003). Similarly, pre-judgment retrieval fluency (that is, its latency and success) correlates with metacognitive judgments about past and future memory performance (Kelley and Lindsay, 1993; Matvey et al., 2001; Dougherty et al., 2005; Koriat and Ma'ayan, 2005).

Therefore, it seems that, contrary to the “trace-strength” and “direct translation” hypotheses, the results of post-decisional and post-response processing could be integrated into metacognitive judgments. However, this additional information would be fed only into retrospective reports, making them more accurate than prospective ones. In order to test this hypothesis we designed a task in which participants rated their decision certainty either immediately before or immediately after a type 1 response. The main task required participants to work on anagrams for a short period of time, and then to decide whether a presented target word was a solution. The target word was either a solution or was matched to the solution in a way that rejecting it as a potential solution was not easy. On the contrary, targets were often misleading because they contained almost the same letters as the anagrams. To minimize the possibility of participants counting, remembering and comparing the letters between anagrams and targets, at least seven letter anagrams were used and the targets were presented briefly. There was one condition with a retrospective confidence report (target-decision-metacognition, tDM). In this condition participants first saw a target, then responded regarding whether or not the target was an anagram solution and then rated their confidence in the preceding response. Two conditions were created for prospective confidence reports in which participants were asked to rate their certainty in the following response. Prospective conditions differed from each other in respect to whether confidence ratings were given after or before seeing a target word. In the metacognition-target-decision condition (MtD) participants first rated their confidence in a future response and were then presented with a potential solution. In the target-metacognition-decision condition (tMD) participants were first presented with a potential solution and then rated confidence in a future response. The two prospective conditions were introduced in order to control possible problems caused by targets being presented either before or after a metacognitive judgment. In the tMD condition, when the target is presented before confidence ratings one cannot exclude the possibility that participants make their decisions covertly before reporting their confidence. On the other hand, when participants are required to rate their future decision certainty before seeing the target (MtD), they are provided with less decision-related information. We hypothesized that metacognitive accuracy would be lower in both prospective conditions than in the retrospective condition. This would support the idea that different internal cues are integrated in each type of judgment and this difference is not explained simply by the amount of information provided by the task.

## Methods

### Participants

Ninety-seven volunteers, 65 women, aged 18–30 (*M* = 21.73, *SD* = 2.1) took part in the experiment in return for a small payment. All participants had normal or corrected to normal vision and gave written consent to participation in the study. The ethical committee of the Institute of Psychology approved the experimental protocol.

### Materials

The experiment was run on PC computers using E-Prime. For the purpose of the study, 60 three-syllable Polish nouns containing 7–10 letters were chosen from a frequency list (Mandera et al., 2015). The words were paired so that 19 pairs of them differed by just 1 letter (in English this could be: SENATOR-TOASTER) and 11 pairs, which differed by 2 letters (e.g., RESTAURANT-TRANSLATOR), which could be either exchanged or added. The anagrams were made by randomly mixing the letters of one word in a pair. Three judges chose one letter string for each anagram that was least similar to any word and did not contain any syllables included in the solution or target word. The list of anagrams to solve was the same for all participants but different solutions (i.e., correct or incorrect) were suggested. Participants were asked to rate the confidence of their future or past decision (“How confident are you that you will make the right decision?” or “How confident are you that you made the right decision?”). The options were: “I am guessing,” “I am not confident,” “I am quite confident,” and “I am very confident.”

### Procedure

Participants were tested in small groups in a computer laboratory and randomly assigned to one of three conditions: they firstly decided if a target word presented on a screen was an anagram solution and then judged their confidence (target-decision-metacognitive judgment, tDM), they prospectively rated the confidence of their decision after seeing a target (tMD), or they prospectively rated the confidence of their decision before seeing a target (MtD).

**Figure 1:**
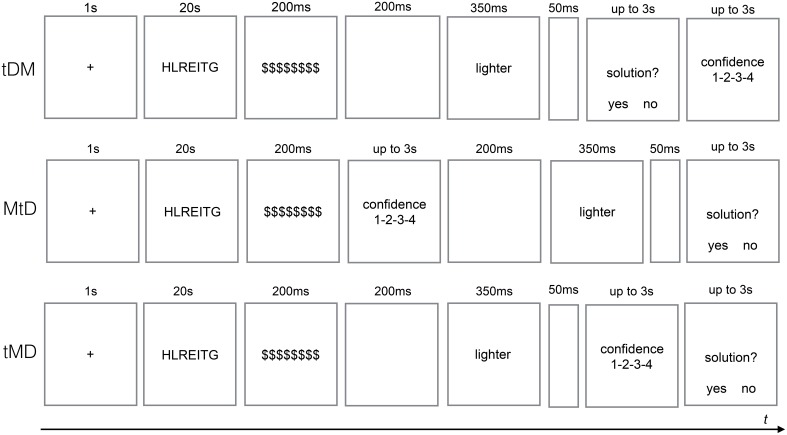
**The three conditions of the anagram task**. tDM, target-decision-metacognitive judgment; MtD, metacognitive judgment-target-decision; tMD, target-metacognitive judgment-decision.

The outline of the procedure is presented in Figure 1. Each trial started with a fixation-cross appearing for 1 s and followed by an anagram written in capital letters. Participants had 20 s to work on each anagram. Then it was masked by $$$$$$$$$$ symbol for 200 ms. In the tDM condition, a 200 ms blank screen followed the mask and then a target word appeared for 350 ms. Then a word “solution?” was presented in the center with two options “yes” and “no” on both sides. After making the decision participants were asked to rate their certainty on the confidence scale. In the MtD condition participants were first asked to rate their confidence in correct decision about the solution, then they were presented a target word (preceded by the mask and the blank screen), with decision to be made at the end of a trial. In the tMD condition a target word (preceded by the mask and the blank screen) were presented first and then participants were asked to rate the certainty of the solution decision and, in the end, to make the actual decision. Participants had 3 s for decision and metacognitive judgment. Decision about a target word was expressed by pressing “1” or “2” key on a numerical keyboard with the right hand, and the confidence level was reported with keys “1,” “2,” “3,” and “4” using the left hand (from “1” representing “I am guessing” to “4” representing “I am very confident”). Half of the presented targets were the correct solutions. There were two blocks of trials with 15 anagrams each. At the beginning of experiment participants were shown two examples of simple anagrams and had a chance to try to solve them.

### Data analysis

Metacognitive accuracy was operationalized as the relationship between the accuracy of identifying an anagram solution and the reported confidence in this decision (Sandberg et al., 2010; Norman et al., 2011). The relation between confidence and accuracy was analyzed using logistic regression, which is the correct model for predicting binary outcomes like accuracy (Norman and Price, 2015). Logistic regression analysis is free from theoretical assumptions about the source of confidence and therefore is considered by us as a better method of analysing metacognitive accuracy than the popular alternatives based on signal detection theory (but see: Rausch et al., 2015). There are few other important advantages of logistic regression that are worth mentioning: (1) it does not require binary metacognitive ratings, therefore it does not force us to simplify the model, (2) the mixed model framework allows us to answer several statistical questions as well as to control for the random effect of subjects in the context of a single comprehensive analysis, (3) mixed models tolerate unbalanced designs.

The mixed logistic regression models were fitted using the lme4 package in the R Statistical Environment (Bates et al., 2015; R Core Team, 2015) using standard (0/1) contrast coding. In our main model the fixed effects were Confidence ratings (4 levels), Condition (3 levels) and their interaction, and the only random effect included was the participant specific intercept. Confidence ratings were centered on the lowest values (guessing) and the basic condition was the retrospective judgment condition (tDM). Therefore the regression slope reflects the relation between metacognition and accuracy (metacognitive accuracy) while the intercept informs about performance level when participants report guessing. Statistical significance was assessed by means of the Wald test.

It was important to test metacognitive accuracy of only those participants who were actually working on solving anagrams and did not simply guess whether a presented word was or was not the solution. Since it is hard to keep performance in a problem-solving task on a constant and higher than chance level, we decided *a priori* to analyse the data of only those participants whose performance in the anagram task would be equal or higher than 60%. We also fitted the model to the data of all participants with performance above 50% obtaining similar coefficients and pattern of results but due to aforementioned reasons, with the weaker effects.

## Results

**Figure 2:**
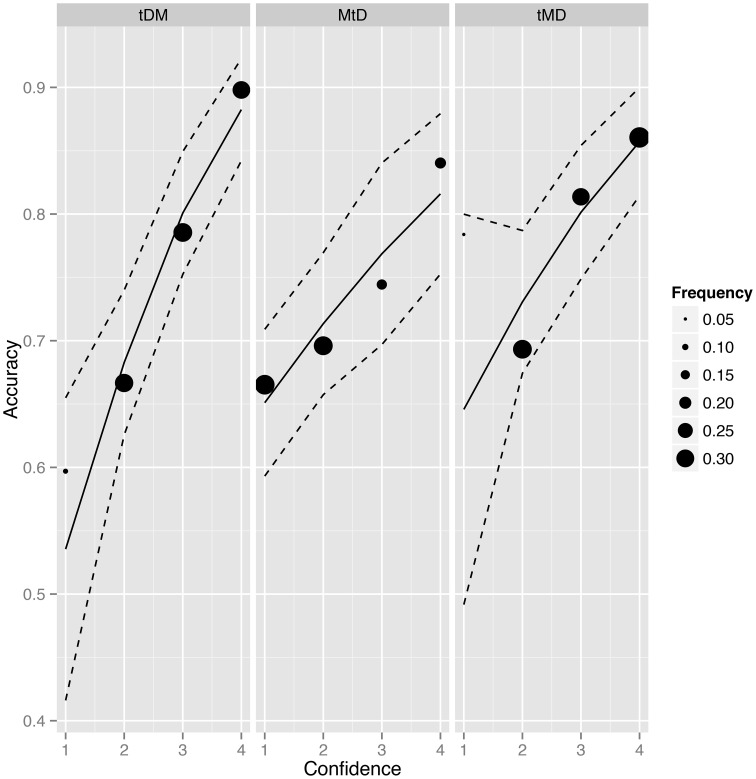
**Average accuracy, scale response frequency and model fit for the relationship between accuracy and confidence ratings in each condition (tDM, target-decision-metacognition; MtD, metacognition-target-decision; tMD, target-metacognition-decision)**. The position of filled circles represents average accuracy for each scale point. The frequency describes the proportion of each confidence rating response in each condition. Dashed lines represent 95% confidence intervals.

Participants missed their responses in six per-cents of trials. Accuracy level in the anagram task for each condition equalled: tDM—70%, tMD—72%, MtD—66% and was significantly lower in MtD than in other conditions (tDM-MtD: *z* = 2, *p* < 0.05; tMD-MtD: *z* = 2.9, *p* < 0.01). The data of 10 participants with accuracy level below 60% and 1 participant with confidence rating variance equal to 0 were excluded from the analysis. Among the remaining participants accuracy differed only between tMD and MtD condition (*z* = 2.6, *p* < 0.01). We did not find significant differences between conditions in terms of decision bias, therefore the “yes” and “no” response were chosen equally frequently (|z| ≤ 1, *p* > 0.3). The descriptive statistics of task accuracy and confidence ratings are presented on Table 1 and Figure 2.

**Table 1:** **The average accuracy and confidence in each condition**.

	***n***	**Accuracy (%)**	**Confidence**	
			***M***	***SD***
tDM	30	73	2.83	0.95
MtD	28	74	2.20	1.09
tMD	28	71	2.93	0.92

In order to analyse the effect of decision-metacognition order on metacognitive accuracy we fitted a mixed logistic regression model to Accuracy data with Condition (tDM, tMD, MtD), Confidence and their interaction as fixed effects, and a random participant specific intercept (Table 2 and Figure 2). The first row in Table 2 (intercept) estimates the average accuracy (on the logit scale) in the baseline condition (condition tDM, lowest rating = guessing). When reporting guessing, participants' accuracy in tDM did not differ from chance level (*z* = 0.85, *p* = 0.4). The second and the third row show that the decision accuracy for the lowest scale rating was not significantly different in tMD than in tDM (*z* = 1.78, *p* = 0.08) but was significantly better in MtD than in tDM (*z* = 2.35, *p* = 0.02). The forth row estimates the relationship between Accuracy and Confidence in the tDM condition, and shows it is statistically significant (*z* = 6.73, *p* < 0.001). The fifth row indicates that relation between Accuracy and Confidence was higher when the confidence was rated retrospectively than when the confidence was rated prospectively but after target word presentation (the difference between tDM and tMD slopes: *z* = −1.68, *p* = 0.046, directional test). Similarly, the last row shows that metacognitive accuracy was stronger when confidence was rated retrospectively than when the confidence was rated prospectively but before target word presentation (the difference between tDM and MtD slopes: *z* = −2.77, *p* = 0.003, directional test). Reparametrisation of the model showed no difference in Accuracy-Confidence relation between MtD and tMD conditions (*z* = −0.9, *p* = 0.4). We also calculated the correlation between Confidence ratings and Accuracy within each condition; it was statistically significant for all of them (tDM: *z* = 6.73, *p* < 0.001; tMD: *z* = 4, *p* < 0.001; and MtD: *z* = 3.7, *p* < 0.001).

**Table 2:** **Regression coefficients for the logistic regression mixed model for accuracy**.

**Number of participants: 86**	**Estimate**	***SE***	***Z***	***p***
**Number of observations: 2368**				
Intercept	0.14	0.17	0.85	0.40
Condition tMD	0.46	0.26	1.78	0.08
Condition MtD	0.48	0.21	2.35	0.02^*^
Confidence	0.63	0.09	6.73	< 0.001^***^
Confidence: Condition tMD	−0.23	0.14	−1.68	0.046^*^
Confidence: Condition MtD	−0.34	0.12	−2.78	0.003^**^

*likelihood ratio χ${}_{\left(5\right)}^{2}=$ 89, p < 0.001*.

* p < 0.05;

** p < 0.01;

*** *p < 0.001*.

In order to explore the possibility that the effect of condition could have been caused merely by accumulation of semantic activation of an anagram and target that was its actual solution, we created a variable “Target is solution.” When targets were anagrams' solutions the average accuracy was 76% and the mean level of confidence was 2.6 (*SD* = 1). When targets were not solutions, the average accuracy equalled 75% and the confidence level was 2.7 (*SD* = 1.08). After fitting a model that included this variable (Condition × Confidence rating × Target is solution) we did not observe any additional significant effects and the model fit did not differ [model's fit comparison: χ${}_{\left(6\right)}^{2}=7$, *p* = 0.3].

Additionally, we analyzed how the task condition influenced metacognitive judgments themselves. Therefore we compared the frequency of high and low confidence ratings between conditions. All the ratings were encoded as binary outcomes, either high (“I am quite confident,” “I am very confident”) or low confidence (“I am guessing,” “I am not confident”). Mixed logistic regression analysis revealed that high ratings were given significantly more often when participants saw the target word before the metacognitive judgment (the difference between MtD and tDM: *z* = −4, 5, *p* < 0.001 and the difference between MtD and tMD: *z* = 5, *p* < 0.001). We did not find any difference between tMD and tDM (when the target was always shown first) in the frequency of high confidence ratings (*z* = 0.6, *p* = 0.6).

## Discussion

The goal of the experiment was to find out whether prospective and retrospective metacognitive judgments differ in their accuracy, and more specifically, whether confidence in response is more accurate once the response has been given. On the theoretical level our study aimed to differentiate between two views on the source of metacognitive judgments, that is whether they are based on the same or different information than type 1 responses. The results of the experiment showed that both retrospective and prospective confidence judgments correlated with performance level, but confidence ratings were less accurate when given prospectively. Therefore although both types of judgments seem to be partially based on the same information as type 1 responses (in that they are both related to performance), different factors influence their accuracy. One of the prospective conditions (MtD) differed from the retrospective one also in the level of the anagram task performance for the lowest scale point. This means that participants in MtD condition performed better that in tDM when reporting guessing. The results of the experiment also showed the effect of tasks on confidence rating strategy. Participants who were not shown the suggested solution before metacognitive judgment was required were less confident in their decisions.

The results supported our hypothesis that retrospective confidence judgments would be more accurate than prospective judgments and are in line with the view that metacognitive reports are based on different information than the decisions they relate to. One controversy between metacognitive theories is whether confidence is built only on evidence available at the moment of a type 1 decision (Vickers and Lee, 1998; Higham et al., 2009) or whether it is the result of a separate evidence accumulation stage that happens after the primary decision has been made (Petrusic and Baranski, 2003; Pleskac and Busemeyer, 2010). If type 1 response and metacognition are based on the same information, it means that in case of prospective judgment a decision or memory access attempt has to be made at the time of the judgment. This could result in lower performance level due to the smaller amount of time available for processing decision-related evidence, but without changing metacognitive accuracy. However, the stronger relationship between retrospective reports and task accuracy suggest that prospective judgments are deprived of additional information that might increase the accuracy of retrospective judgment.

In our opinion, the results add to the body of evidence from neurophysiological and behavioral experiments, as well as from modeling, suggesting that metacognitive judgments could be based on evidence unavailable to a type 1 response (Petrusic and Baranski, 2003; Ploran et al., 2007; Resulaj et al., 2009; Hilgenstock et al., 2014; Graziano et al., 2015). Moreover, the results suggest that response-related information could be integrated into metacognitive judgment. A variety of studies in different paradigms has shown a negative correlation between reaction time and metacognitive rating (Kelley and Lindsay, 1993; Matvey et al., 2001; Petrusic and Baranski, 2003; Dougherty et al., 2005; Koriat and Ma'ayan, 2005; Mealor and Dienes, 2013). Although it could be argued that reaction time only reflects the quality of stimulus or memory trace, Kiani et al. (2014) have recently shown the same relation for fixed stimulus strength. One interesting direction of investigating metacognition is the potential link to other monitoring functions such as error detection (Scheffers and Coles, 2000; Steinhauser and Yeung, 2012; Boldt and Yeung, 2015; Graziano et al., 2015) or interoceptive feedback (Wessel et al., 2011). A recent study has also shown that metacognitive judgment could be influenced by motor-related neural activity. In the experiment by Fleming et al. (2015) confidence was lowered by transcranial magnetic stimulation of the premotor cortex area associated with a response to an item that had not been chosen. Although most of the aforementioned data come from studies on perception, the process of building metacognitive judgment about memory-based problem solving performance might be even more complex.

Apart from the overall metacognitive accuracy, prospective judgments also differed from retrospective ones in terms of the average task performance for the lowest scale point that is when participants reported guessing. Moreover, participants in the MtD condition who were required to rate their decision certainty before seeing a potential solution more often reported low confidence. There are at least two possible interpretations of those results. Firstly, it could indicate that participants used a more cautious strategy when assessing their certainty before responding to a type 1 task and were even more careful when having to do so before even seeing a target. Therefore, both above chance level accuracy for the lowest confidence rating and high frequency of low ratings would indicate cautious strategy. The other interpretation states that stimulus-related information might not always be sufficient to reduce basic uncertainty, and confidence arises when more internal and external cues are available. This could especially be the case for the MtD condition, for which not knowing the target before a metacognitive judgment was required made participants uncertain about their future response accuracy.

One potential problem with comparing metacognitive accuracy between the three conditions is the difference in type 1 task performance level. Participants in the MtD condition who were required to assess their confidence in a future decision before seeing the target words performed the anagram task worse than the other groups. Although task difficulty might affect both the decision and metacognitive judgment (but see: Scott et al., 2014 for “blind insight” effect), we found no differences in metacognitive accuracy between the two prospective conditions even though they differed in task 1 performance level. A probable explanation of the lower performance in the MtD condition is that the anagram-related information in memory was fading because in this condition the time between solving the anagram and seeing the target was longest. This indirectly supports our prediction that in this task participants make decisions at the moment of seeing the target and then just remember their “yes” or “no” response until they express it. If this were true, it would mean that not only decision but also motor response should be considered as factors increasing metacognitive judgment accuracy.

To sum up, the results of our experiment showed that confidence judgments are more accurate when they refer to the response already given than when they are about a future response. Our results seem to be inconsistent with “direct translation” hypothesis and fit better within a dynamic, two-stage evidence accumulation framework which takes into consideration both time and post-decision processing (e.g., Pleskac and Busemeyer, 2010). However, we think that there is a need for an extended theory of confidence accommodating data suggesting that confidence is a result of monitoring the entire decision-making process (e.g., Graziano et al., 2015) and could be informed by many sources of evidence. Also, although studies on metamemory and confidence judgments describe different time scales and different levels of processing, we believe more work should be done to integrate the knowledge from those fields. As confidence measured in both research paradigms refers to the same judgment about one's performance this integration would be fruitful for better understanding the mechanisms of human metacognition (Fleming and Dolan, 2012; Yeung and Summerfield, 2012). For example, an evidence accumulation framework should attempt to explain all types of judgments, including JOLs and FOKs, and to specify what types of evidence could be accumulated to each of them.

In the end we would like to point out that our results might also have methodological and theoretical implications for consciousness research. Confidence ratings and other metacognitive scales (also called type 2 judgments) have been widely used in this area (for a review see Timmermans and Cleeremans, 2015) as tools for discriminating between conscious and unconscious knowledge (Dienes et al., 1995) and for assessing the level of stimuli awareness (Sandberg et al., 2010). The results of our experiments are in line with data showing that when using subjective scales (like confidence rating or Perceptual Awareness Scale) one should take into account the order of type 1 and type 2 responses (Wierzchoń et al., 2014). Although in implicit learning studies type 2 judgments are usually given after the primary forced-choice response, this is not always the case for perception studies (Del Cul et al., 2007, 2009; Rounis et al., 2010; Wierzchoń et al., 2014; Jachs et al., 2015). From a theoretical point of view, it is worth trying to reinterpret the discussion between the one-source and the multi-source views on metacognition in the context of consciousness theories. In consciousness research, if one discusses the issue it is usually assumed that the awareness judgment is based on the availability of a representation of information that is becoming available (e.g., Del Cul et al., 2009). This assumption seems to be consistent with one-source view on metacognition. A similar assumption is made when metacognitive awareness is assessed as the meta-d' measure used in context of those studies assumes that judgments refer to the representation of the stimuli. Our data seem to suggest that the accumulation models should be discussed in the context of theories of consciousness. This seems most obvious in the context of hierarchical models of consciousness (Lau and Rosenthal, 2011; Timmermans et al., 2012). These models claim that conscious awareness of a given stimulus or memory content requires the completion of processes leading to a type 1 decision in order to re-represent their content. Our data suggests that this re-presentation could take into the account not only the stimuli representation but also that some other information contributing to type 2 might be involved. Thus, we believe that establishing a connection between hierarchical models of consciousness and metacognition might be a fruitful way to research metacognitive awareness.

## Author contributions

MS and MW proposed the concept of the study. MS collected data, and BP made statistical analysis, with suggestions provided by all co-authors. MS drafted the manuscript; MW and BP provided critical revisions. All authors approved the final version of the manuscript for submission.

## Funding

This work was supported by the National Science Centre, Poland HARMONIA grant given to MW (2014/14/M/HS6/00911).

### Conflict of interest statement

The authors declare that the research was conducted in the absence of any commercial or financial relationships that could be construed as a potential conflict of interest.
